# The association of kidney function with repetitive breath-hold diving activities of female divers from Korea, Haenyeo

**DOI:** 10.1186/s12882-017-0481-1

**Published:** 2017-02-23

**Authors:** Yun Jung Oh, Ji Yong Jung, Sung Soo Kim, Kyong-Suk Chae, Jiwon Rhu, Chungsik Lee

**Affiliations:** 1 Department of Internal Medicine, Cheju Halla General Hospital, Doryeong-ro 65, Jeju, 63127 Korea; 2 Department of Internal Medicine, Gachon University Graduate School of Medicine, Incheon, Korea; 3 Department of Internal Medicine, Gachon University School of Medicine, Incheon, Korea; 4 Department of Internal Medicine, Division of Nephrology, Gachon University Gil Medical Center, Incheon, Korea

**Keywords:** Breath-hold diving, Kidney function, Repetitive apnea

## Abstract

**Background:**

Voluntary apnea during breath-hold diving (BHD) induces cardiovascular changes including bradycardia, reduced cardiac output, and arterial hypertension. Although the impacts of repetitive BHD on cardiovascular health have been studied previously, the long-term risk for kidney dysfunction has never been investigated.

**Methods:**

A cross-sectional propensity score-matched study was performed to evaluate the influence of repetitive long-lasting BHD on kidney function. Using matching propensity scores (PS), 715 breath-hold female divers (Haenyeo) and non-divers were selected for analysis from 1,938 female divers and 3,415 non-divers, respectively. The prevalence of chronic kidney disease (CKD) defined as an estimated glomerular filtration rate (eGFR) calculated to be less than 60 ml/min/1.73 m^2^ was investigated in both diver and non-diver groups.

**Results:**

The prevalence of CKD was significantly higher in breath-hold divers compared with non-divers after PS matching (12.6% vs. 8.0%, *P* = 0.004). In multivariate analysis, BHD activity was significantly associated with the risk of CKD in an unmatched cohort (OR, 1.976; 95% CI, 1.465–2.664). In the PS-matched cohort, BHD remained the independent risk factor for CKD even after adjusting for multiple covariates (OR 1.967; 95% CI, 1.341–2.886).

**Conclusion:**

Shallow but repetitive intermittent apnea by BHD, sustained for a long period of time, may potentially cause a deterioration in kidney function, as a long-term consequence.

**Electronic supplementary material:**

The online version of this article (doi:10.1186/s12882-017-0481-1) contains supplementary material, which is available to authorized users.

## Background

Apnea, whether voluntary or involuntary, induces several physiological changes, which are a type of protective responses to hypoxia and involve potential health hazards as well. Voluntary apnea during breath-hold diving (BHD) induces cardiovascular adaptations characterized by bradycardia with reduced cardiac output and peripheral vasoconstriction with increased blood pressure (BP), which are together currently known as the human diving response [1–6]. Bradycardia is elicited by increased vagal activity, and peripheral vasoconstriction is related to increased sympathetic activity [4, 5, 7]. The pronounced bradycardia during BHD reduces oxygen consumption by the heart, and selective vasoconstriction mainly redistributes decreased cardiac output to the heart and brain, which are the most sensitive to hypoxia, making other tissues more likely to be exposed to relatively greater levels of hypoxia [8, 9].

Excessive hypoxia due to extreme BHD activity can be very dangerous and can lead to serious acute health problems, such as pulmonary edema and hemorrhage, cardiac arrhythmias, blackout, decompression sickness, and even death [4, 5]. However, although acute cardiovascular changes induced during BHD and fatal complicaitons by extreme diving have been studied, the long-term effects of shallow but frequent intermittent apnea by repetitive BHD have not been well studied. Based on the aforementioned cardiovascular diving response, there is a possibility that even shallow but prolonged repetitive apnea by BHD may cause negative health consequences such as cardiovascular, respiratory, and cerebrovascular disease. Obstructive sleep apnea (OSA) is a condition in which breathing stops involuntarily for a short time during sleep, has something in common with BHD in terms of the repeated exposure to intermittent apnea-induced hypoxia, even though it’s pathophysiology is different from what of BHD. The cardiovascular complications of OSA were shown in previous studies [10–13] and the chronic intermittent hypoxia was suggested as one of the mechanisms responsible for sympathetic activation and increased cardiovascular morbidity in OSA [10, 14, 15]. Therefore, the notion that breath-hold divers are exposed to chronic intermittent hypoxia, similar circumstance to patients with OSA, may raise a possibility that BHD may potentially predispose breath-hold divers to long-term health risks by exposing them to chronic frequent hypoxic conditions. Furthermore, as kidney function and cardiovascular disease are closely associated [16–18] and BHD induces cardiovascular changes, we hypothesize that renal impairment may be one of the potential long-term health risks of repetitive BHD.

Korean female divers, called Haenyeo, are professional breath-hold divers who have traditionally harvested marine products on Jeju island. They are engaged in daily shallow short dives down to an average of 5–10 m of sea water, holding their breath for an average 30 s [19, 20]. These Korean female divers have kept their diving regimen for more than 2000 years [4, 5, 19]. These female divers belong to the Haenyeo Union, which is attached to the Fishery Cooperative Union, and work in accordance with the laws and regulations of the association. According to a report by the Union, approximately 9,900 female divers are current members, and approximately 4,600 women are at present engaged in diving work with an average career of 40 to 50 years. Therefore, they are relevant subjects for evaluating the long-term consequences of frequent BHD on kidney function. We aimed to evaluate the effects of repeated, voluntary, and long-lasting intermittent apnea on kidney function in the population of Korean female breath-hold divers.

## Methods

### Ethics approval and consent to participate

The present study was approved by the institutional review board (IRB) of Cheju Halla General Hospital (2013-M05) and performed in accordance with the principle of Helsinki Declaration. This work was a cross sectional and non-interventional study, therefore written informed consent was waived after approval of the IRB.

### Study population

This study was a cross-sectional propensity score (PS)-matched study to investigate the effect of repeated voluntary BHD on kidney function. We conducted this study using data collected from diving women (Haenyeo) and non-diving women who were the inhabitants of Juju island and visited Cheju Halla General Hospital (Jeju, Korea) for health check-ups. For the study, diving and non-diving women who presented to Cheju Halla General Hospital between November 2007 and December 2012 were screened and whether the woman was a diver (Haenyeo) or not was distinguished by presence of registered Haenyeo membership number issued by the governor of Jeju province. In cases where the subject had taken checkup repeatedly for the period, only the first visit data were included. Initially, we recruited 3,679 women divers and 4,237 non-divers, who were aged ≥ 20 years old and underwent voluntary health screenings. From that group, we excluded 1,682 subjects with missing serum creatinine data and 881 subjects who had insufficient information about co-morbidities or comparable laboratory data apart from their serum creatinine levels. A total of 5,353 participants, who were composed of 1,938 female divers and 3,415 non-divers, were included in the final analysis.

### Study variables

Demographic and clinical data were obtained from a review of medical records. We obtained demographic data, including age, sex, and medication history. Information about medical co-morbidities such as diabetes, hypertension, and cardiovascular disease was also collected. Cardiovascular disease included coronary artery disease and cerebrovascular disease. Laboratory parameters included serum creatinine, hemoglobin, albumin, uric acid, total cholesterol, calcium, phosphorus, and alkaline phosphatase (ALP) levels. Measurement of serum creatinine was performed using the kinetic Jaffe method. The estimated glomerular filtration rate (eGFR) was calculated using the Chronic Kidney Disease Epidemiology Collaboration (CKD-EPI) [21, 22]. CKD was defined as an eGFR that was less than 60 ml/min/1.73 m^2^.

### Statistical analysis

Because there were substantial differences in baseline characteristics between the diver and non-diver groups, we used PS matching to reduce potential confounding and selection biases and to achieve balance. The PS is the conditional probability of receiving a particular exposure (BHD activity) given a vector of baseline measured covariates [23–25]. We estimated propensity scores for being a female diver (performing BHD activity) for all subjects using a non-parsimonious multivariate logistic model [26, 27]. A multivariate logistic model was constructed to predict the probability of being a female diver given the following covariates: age, diabetes, hypertension, cardiovascular disease, hemoglobin, albumin, total cholesterol, uric acid, calcium, phosphorus, and ALP. Using these covariates, a PS was calculated for each individual. We then used the estimated PS to match 715 female divers with 715 non-divers at a ratio of 1:1 using the Greedy matching algorithm [28]. The C-statistic for the logistic model was 0.936 according to the receiver operating curve, indicating a high degree of discrimination. Standardized differences were estimated before and after matching to assess the balance in baseline covariates between the two groups [29, 30]. After PS matching, standardized differences for a given covariate were reduced below 10%, indicating substantial improvement in the balance of covariates between the groups. For the matched cohort, paired comparisons were performed using a paired *t*-test and the McNemar test for continuous and categorical variables, respectively. We performed PS matching with the SAS software package (SAS Institute, version 9.3, Cary, NC).

Continuous variables are presented as the mean ± SD and were compared using Student’s *t*-test. Categorical variables are expressed as frequencies or percentages, and comparisons between groups were performed using the chi-squared test or Fisher’s exact test, as appropriate. Multivariate logistic regression analysis was performed to identify the independent effect of BHD activity on the risk of CKD. Two-sided *P* values were reported, and statistical significance was defined as *P* < 0.05. All analyses were performed using SPSS Statistics software (version 21.0, Chicago, IL), except PS matching.

## Results

A total of 7,916 women who had presented for health check-up were screened, and 5,353 women with sufficient available data were included in this study. Of the 5,353 women, 1,938 subjects were divers (Haenyeo), and the other 3,415 subjects were non-divers. Table 1 shows the demographic and clinical characteristics of the study population. Prior to PS matching, there were considerable differences in baseline covariates between the female diver and non-diver groups. The mean age of female divers (67.2 ± 9.3) was greater than that of non-divers (45.7 ± 12.2). The prevalence of hypertension and cardiovascular disease was higher in the diver group compared with non-diver women, but the prevalence of diabetes was not different between the groups. The female divers were more likely to have lower levels of hemoglobin and serum albumin than non-diver females. The levels of uric acid, total cholesterol, calcium, and ALP, were higher in the female-diver group compared with the non-diver group.

**Table 1:** Clinical characteristics of study participants

	Before Matching				After Propensity Matching			
	Non-divers	Female divers	*P*	Standardized differences	Non-divers	Female divers	*P*	Standardized differences
	(*n* = 3,415)	(*n* = 1,938)			(*n* = 715)	(*n* = 715)		
Age	45.7 ± 12.2	67.2 ± 9.3	<0.001	2.646	61.4 ± 10.4	60.9 ± 8.7	0.140	0.060
Diabetes	205 (6.0%)	140 (7.2%)	0.080	0.048	58 (8.1%)	53 (7.4%)	0.685	0.026
Hypertension	43 (1.3%)	609 (31.4%)	<0.001	0.891	39 (5.5%)	53 (7.4%)	0.109	0.077
Cardiovascular disease	89 (2.6%)	342 (17.6%)	<0.001	0.514	45 (6.3%)	45 (6.3%)	1.000	0.000
Hemoglobin (g/dL)	12.9 ± 1.1	12.8 ± 1.3	0.024	0.030	13.0 ± 1.1	13.0 ± 1.2	0.458	0.016
Serum albumin (g/dL)	4.4 ± 0.3	4.2 ± 0.4	<0.001	0.159	4.3 ± 0.3	4.3 ± 0.3	0.464	0.008
ALP (IU/L)	64.8 ± 20.9	85.3 ± 42.5	<0.001	0.669	78.5 ± 24.1	78.3 ± 24.4	0.844	0.013
Uric acid (mg/dL)	4.7 ± 1.0	4.9 ± 1.5	<0.001	0.102	4.8 ± 1.2	4.8 ± 1.4	0.729	0.012
Total cholesterol (mg/dL)	189 ± 34	196 ± 40	<0.001	0.269	202 ± 38	200 ± 37	0.275	0.076
Calcium (mg/dL)	8.9 ± 0.5	9.0 ± 0.6	<0.001	0.029	9.0 ± 0.5	9.0 ± 0.6	0.324	0.013
Phosphorus (mg/dL)	3.7 ± 0.5	3.6 ± 0.6	<0.001	0.041	3.7 ± 0.5	3.7 ± 0.6	0.930	0.002

Continuous variables are expressed as the mean ± SD, and categorical variables are expressed as the number (percentage). *Abbreviations*: *ALP* alkaline phosphatase

**Fig. 1:**
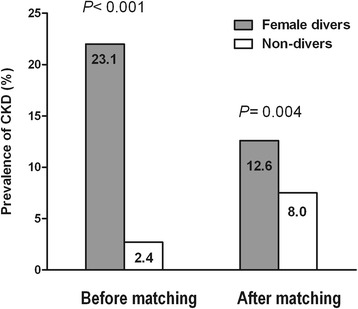
Prevalence of chronic kidney disease (CKD) before and after propensity score (PS) matching in women divers (*gray* column) and non-divers (*white* column). CKD was still more prevalent in Female divers than non-divers after PS matching (12.6% vs 8.0%; *P* = 0.004)

Using PS matching, 1,430 women with similar PS scores were identified and 715 women divers were successfully matched to non-divers, which was indicated by a standardized difference <10% between the two groups. After matching, there were no significant differences in covariates between the female divers and non-divers. However, CKD was significantly more prevalent in the female diver group compared with the non-diver group, even in the matched cohort (12.6% vs. 8.0%, *P* = 0.004; Fig. 1). When kidney function was classified into three groups using categorized eGFR groups (eGFR ≥ 60 ml/min/1.73 m^2^, 45 ml/min/1.73 m^2^ ≤ eGFR < 60 ml/min/1.73 m^2^; early CKD, and eGFR < 45 ml/min/1.73 m^2^; late CKD), the proportions of early CKD as well as late CKD significantly increased in female divers compared with non-divers in the unmatched (*P* < 0.001) and matched cohort (*P* = 0.009) (Additional file 1: Figure S1).

We investigated whether BHD was significantly associated with the prevalence of CKD, using a logistic regression model. An unadjusted analysis revealed that in the female diver group, age, diabetes, hypertension, and cardiovascular disease were associated with a significant increase in CKD prevalence. The levels of serum albumin and hemoglobin showed an inverse relationship with the prevalence of CKD. After adjusting for multiple covariates, including age, diabetes, hypertension, cardiovascular disease, hemoglobin, serum albumin, and total cholesterol, BHD activity was significantly associated with the risk of CKD (Table 2). Female divers had a nearly 2-fold increased risk of CKD compared with non-diver women in the unmatched cohort (OR, 1.976; 95% CI, 1.465–2.664).

**Table 2:** Risk factors for CKD on multivariable logistic regression analysis in the unmatched cohort

	Unadjusted OR (95% CI)	*P*	Adjusted^a^ OR (95% CI)	*P*
Female diver	12.221 (9.583–15.586)	<0.001	1.976 (1.465–2.664)	<0.001
Age	1.144 (1.131–1.157)	<0.001	1.129 (1.115–1.143)	<0.001
Diabetes	2.517 (1.903–3.328)	<0.001	1.586 (1.129–2.227)	0.008
Hypertension	5.503 (4.500–6.730)	<0.001	1.601 (1.233–2.080)	<0.001
Cardiovascular disease	3.728 (2.938–4.729)	<0.001	0.937 (0.698–1.258)	0.665
Hemoglobin (g/dL)	0.883 (0.822–0.948)	0.001	0.945 (0.867–1.030)	0.201
Serum albumin (g/dL)	0.230 (0.182–0.291)	<0.001	1.143 (0.847–1.541)	0.382
Total cholesterol (mg/dL)	1.005 (1.003–1.008)	<0.001	1.003 (1.000–1.005)	0.069

CKD was defined as an estimated glomerular filtration rate < 60 ml/min/1.73 m2

*Abbreviation*: *OR* odd ratio, *CKD* chronic kidney disease

^a^Adjusted for age, diabetes, hypertension, cardiovascular disease, hemoglobin, albumin, total cholesterol, and breath-holding diving activity

In the PS matched cohort, in which individuals of the diver and non-diver groups were balanced in all covariates, BHD activity remained an independent risk factor of CKD even after adjusting for multiple covariates (Table 3). The OR of female divers over non-divers was 1.967 (95% CI, 1.341–2.886). In addition, when we chose a threshold of eGFR < 45 ml/min/1.73 m^2^ to indicate more advanced CKD, the risk for the advanced CKD was also significantly increased in female divers than non-diver, in unmatched (OR, 3.478; 95% CI, 1.649–7.334; *P* = 0.001) and matched cohort (OR, 3.600; 95% CI, 1.503–8.623; *P* = 0.004) (Additional file 2: Table S1).

**Table 3:** Risk factors for CKD on multivariable logistic regression analysis in the matched cohort

	Unadjusted OR (95% CI)	*P*	Adjusted^a^ OR (95% CI)	*P*
Female divers	1.662 (1.172–2.357)	0.004	1.967 (1.341–2.886)	0.001
Age	1.120 (1.097–1.145)	<0.001	1.125 (1.099–1.151)	<0.001
Diabetes	3.402 (2.131–5.432)	<0.001	1.800 (0.978–3.313)	0.059
Hypertension	2.649 (1.562–4.491)	<0.001	2.418 (1.227–4.766)	0.011
Cardiovascular disease	1.373 (0.729–2.587)	0.326	0.598 (0.287–1.251)	0.173
Hemoglobin (g/dL)	0.913 (0.786–1.061)	0.236	0.931 (0.776–1.117)	0.439
Serum albumin (g/dL)	0.618 (0.356–1.074)	0.618	0.818 (0.434–1.541)	0.533
Total cholesterol (mg/dL)	1.003 (0.999–1.007)	0.183	1.005 (1.000–1.010)	0.050

CKD was defined as an estimated glomerular filtration rate < 60 ml/min/1.73 m2

*Abbreviation*: *OR* odd ratio, *CKD* chronic kidney disease

^a^Adjusted for age, diabetes, hypertension, cardiovascular disease, hemoglobin, albumin, total cholesterol, and breath-holding diving activity

## Discussion

In the present study, conducted on a large number of breath-hold female divers (Haenyeo), we evaluated the health risk of repeated long-lasting BHD activities regarding kidney function. Using PS matching analysis, we found that the prevalence of CKD was significantly higher in breath-hold female divers compared with non-divers. This is the first report showing the association of repeated and long-lasting BHD activities with kidney function. Our result suggests that shallow but repetitive and long-lasting BHD activities have a negative effect on kidney function. Based on these results, we hypothesize that intermittent and repetitive apnea induced by BHD leads to renal impairment as a long-term consequence of persistent BHD activity.

The risks of extreme apnea due to deep BHD, including pulmonary edema and alveolar hemorrhage, blackout, decompression illness, and death, are well known through numerous previous reports. However, the potential risks of shallow but repetitive and long-lasting BHD still remain unknown. The main challenge of BHD is exposure to hypoxic condition and high gas pressure with potential toxic effects [4]. To overcome these challenges, cardiovascular adaptations, including bradycardia, arterial hypertension, and redistribution of blood flow, heave been developed during breath-holding [1–5]. However, despite the physiologic cardiovascular adaptation, there were some reports on the long-term sequelae of BHD, which damaged on neurologic, pulmonary, and cardiovascular system [31–35]. Breath-hold divers are exposed to chronic intermittent hypoxia induced by voluntary apnea, while patients with OSA also encounter intermittent hypoxia by involuntary apnea. Numerous studies regarding OSA suggested that chronic intermittent hypoxia caused chronic sympathetic activation, which eventually led to increase of cardiovascular complications [10, 11, 14, 15]. Therefore, even though the BHD and OSA have different pathophysiology, we may assume that the chronic intermittent hypoxia occurred during BHD also induce the similar subsequent process such as increased sympathetic outflow and resultant cardiovascular complications. In another words, BHD activities for a long-time may potentially make divers prone to the development of cardiovascular and autonomic changes similar to OSA patients. Indeed, peripheral vasoconstriction with increased blood pressure has been demonstrated in both breath-hold divers and OSA patients during apnea, which is associated with increased sympathetic discharge. Most previous studies regarding the health risks of BHD focused on neurological and cardiovascular disease [31, 32, 34–36]. The long-term effect of BHD on kidney function has not been previously investigated. However, considering the argument that the cardiovascular system and kidney function are known to be strongly linked to each other and that the relationship between them is assumed to be bidirectional [16–18], we hypothesize that long-term repeated cardiovascular changes by BHD ultimately influence renal function. This is the only work that has demonstrated that long-lasting repetitive BHD is associated with an increased prevalence of CKD.

Despite the similarities of exposure to intermittent apneic episodes and hypoxia between BHD and OSA patients, there have been conflicting data on the possibility that BHD activities lead to OSA-like cardiovascular complications. One study showed echocardiographic changes indicative of subendocardial ischemia through diving [37], and Scherhag et al. suggested a chronic cardiopulmonary risk of regular BHD based on the early signs of functional cardiopulmonary abnormalities in competitive breath-hold divers [38]. In contrast, another study showed cardiovascular reactivity to intermittent hypoxemia was unimpaired in breath-hold divers, unlike patient with OSA [39], and that repeated episodes of hypoxemia induced by BHD did not increase resting sympathetic activity or blood pressure [40]. However, prior studies relevant to BHD were mostly conducted in young and healthy divers, whereas OSA patients are likely to be adults over middle aged with multiple co-morbidities. Therefore, in addition to the essential different pathophysiology between BHD and OSA, these differences in demographic and clinical characteristics may contribute to the discrepancy of results between these groups. Furthermore, because many previous studies were performed in young divers without long histories of BHD, they could not properly evaluate the long-term health risks of BHD. The present study was mainly conducted in older women who had been exposed to repeated intermittent apnea for considerable periods of time and thus could properly evaluate the long-term consequences of BHD regarding kidney function.

The exact mechanism of long-lasting repetitive BHD activities on kidney function is not fully known. The present study was designed as a cross-sectional research study, and thus, it was unable to establish a mechanism that explains the observed association between kidney function and BHD activities. To our knowledge, this work was the first attempt to investigate kidney function in breath-hold divers. However, cardiovascular changes that develop during BHD may provide a clue to determine the potential mechanism. BHD induced peripheral vasoconstriction with increasing sympathetic activity, which is connected to transient elevation of blood pressure. Thus, we assume that sustained repetitive intermittent hypoxic apnea by long-term BHD may lead to chronic sympathetic activation and hypertension in divers, and hypertension is known as a risk factor for the progression of renal disease. Similarly, in a study of patients with OSA, repetitive intermittent hypoxia during sleep contributed to the sympathetic activation and the development of hypertension [14]. Moreover, several previous studies have shown that a prevalence of CKD was significantly increased in patients with OSA and the severity of nocturnal hypoxia was associated with the rate of decline in kidney function, even after adjustments for relevant multiple covariates [41–44]. These findings may support our speculation that long-term intermittent hypoxia induced by BHD contributes to deterioration of kidney function. In addition, redistribution of blood flow is one diving response, in which blood flow diverts primarily to the heart and brain, which are the most sensitive to hypoxia, while the other organs are more likely to be exposed to a relatively greater extent of hypoxia. Therefore, sustained repetitive BHD may consistently lead to transient renal ischemia and hypoxia, and actually an experimental study has shown that long-term intermittent hypoxia induced renal damage in mice [45]. Breath-hold divers experience transient hyperoxia followed by hypoxia and the effect of intermittent hyperoxia during BHD on endothelial dysfunction has been reported in a previous study [46]. It was hypothesized that intermittent hyperoxia during BHD would increase oxidative stress, in turn, lead to endothelial dysfunction. Indeed, in subsequent study, the researchers demonstrated that oxidative stress markers were increased in repetitive breath-hold divers [47]. Oxidative stress is known to be involved in progression of renal injury [48, 49]. Furthermore, a recent experimental study has shown hyperoxic injury in renal rat tissue [50]. Thus, we may speculate that sustained repetitive intermittent hypoxia and hyperoxia induced by apnea during BHD lead to a long-term risk of renal progression. Shallow but frequent BHD is performed not only by professional commercial divers getting marine products but also by various underwater sport players, including underwater rugby players, hockey players, synchronized swimmers, and recreational divers. Hence, the potential numbers of subjects who are exposed to repetitive intermittent apnea may be larger than previously expected. Nevertheless, there is a lack of data about the long-term health risks of repetitive BHD, especially regarding kidney function. This is the first report suggesting the potential health risk of long-term repetitive BHD regarding kidney function. The results from our work can be used as supporting evidences to improve safety among the BHD population and to decrease health risks.

The strengths of our investigation include a large study population and study subjects with experience in BHD for a significantly long period of time. However, there were several limitations to our study. First, we could not obtain the exact diving exposure time of individuals including the length of time worked as a diver and the information whether the divers involved in this study were currently engaged in diving or no more in diving at that time. However, according to the published data, Korean female divers (Haeneyo) made daily 113–138 dives spending a total of 170–200 min, of which 52–63 min were spent diving submerged and the rest of the time was spent at the water surface [19]. It has been known that the female divers start to dive at midteens and continue their daily diving work until they get to old to dive, usually 70–80 years on average [19, 51]. Therefore, given the reported data, the mean age (67.2 ± 9.3 years) of the study population implies that the present study was conducted in divers with a long-term history of diving and that it could evaluate the long-term effect of BHD on health. Second, there was a lack of information on body mass index (BMI) or muscle mass that could exert on influence on serum creatinine level. Considering somewhat intense physical activity regarding diving work, the difference in BMI between the divers and non-divers is likely to be exist and it may lead to confounding effect on outcome. Third, lower socioeconomic status is known to be one of the important risk factors for CKD. Unfortunately, the data on socioeconomic status of female divers was not available in this study. However, most of Korean female divers earn a living through agriculture as well as diving work, and it is generally accepted that female divers have average economic competence. Thus, given the social perception of the female divers, the overall socioeconomic status of female divers may not be low. Fourth, albuminuria was not included in the definition of CKD. Thus there is the possibility that subjects in early CKD with high-normal GFR and albuminuria could be undetected for this study outcome. Fifth, the current investigation was a cross-sectional study, and therefore, the causality between BHD activity and kidney function could not be inferred. Since the study was performed in subjects with available data, there could be a selection bias. To reduce such selection bias and minimize the difference in baseline covariates between the study groups, we used PS matching analysis. However, there’s still a possibility that differences in unmeasured covariates could lead to biased results. Finally, the present study was conducted in female divers, and thus, our findings is cannot be extended to male divers.

## Conclusions

In conclusion, our study demonstrates a higher prevalence of CKD in female breath-hold divers compared with non-divers. Our results suggest that shallow but repetitive intermittent BHD apnea, sustained for a long period of time, may lead to long-term harmful effects on kidney function. Further studies are warranted to validate our result and establish the mechanisms underlying our findings.

## Additional files

Additional file 1: Figure S1. Proportion of subjects categorized by estimated glomerular filtration rate (eGFR) groups before and after propensity (PS) matching in female divers and non-divers. (TIF 864 kb)
Additional file 2: Table S1. Risk factors for eGFR < 45 ml/min/1.73 m^2^ on multivariate logistic regression analysis in the unmatched and matched cohort. (DOCX 21 kb)

## Abbreviations

ALP: Alkaline phosphatase
BHD: Breath-hold diving
BMI: Body mass index
BP: Blood pressure
CKD: Chronic kidney disease
CKD-EPI: Chronic Kidney Disease Epidemiology Collaboration
eGFR: Estimated glomerular filtration rate
IRB: Institutional review board
OSA: Obstructive sleep apnea
PS: Propensity score
