# Supplementary figures and images for: The association of kidney function with repetitive breath-hold diving activities of female divers from Korea, Haenyeo
Source: BMC Nephrol. 2017 Feb 23;18:75. doi: 10.1186/s12882-017-0481-1 (PMC5322595; doi:10.1186/s12882-017-0481-1)

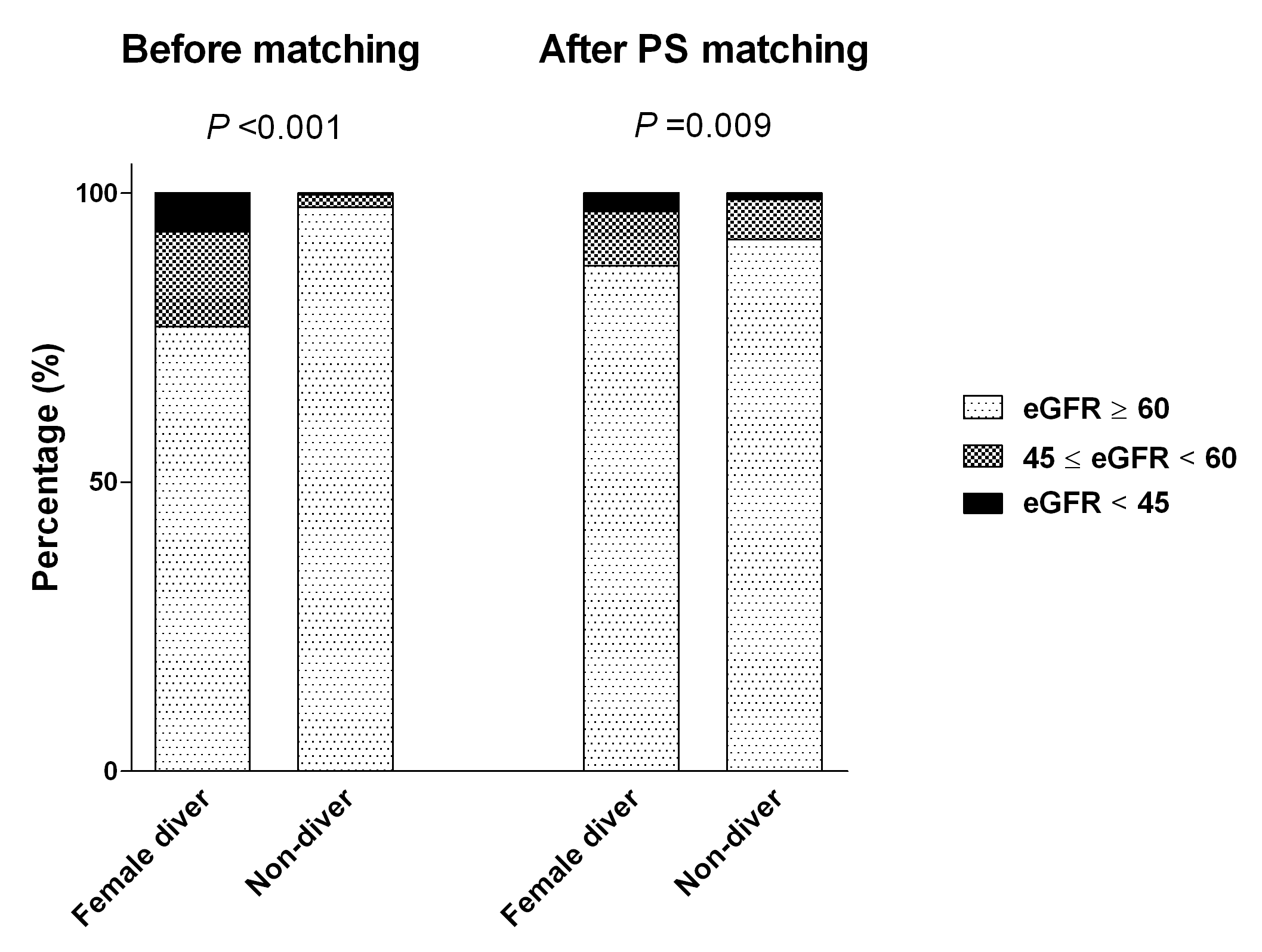

Supplement: Additional file 1: Figure S1. — Proportion of subjects categorized by estimated glomerular filtration rate (eGFR) groups before and after propensity (PS) matching in female divers and non-divers. (TIF 864 kb) [file 12882_2017_481_MOESM1_ESM.tif]
