# Supplementary material for: The association of kidney function with repetitive breath-hold diving activities of female divers from Korea, Haenyeo
Source: BMC Nephrol. 2017 Feb 23;18:75. doi: 10.1186/s12882-017-0481-1 (PMC5322595; doi:10.1186/s12882-017-0481-1)
Supplement: Additional file 2: Table S1. — Risk factors for eGFR < 45 ml/min/1.73 m2 on multivariate logistic regression analysis in the unmatched and matched cohort. (DOCX 21 kb) [file 12882_2017_481_MOESM2_ESM.docx]

**Table S1.** Risk factors for eGFR < 45 ml/min/1.73m^2^ on multivariate logistic regression analysis in the unmatched and matched cohort

|  | Unmatched cohort | | Matched cohort | |
| --- | --- | --- | --- | --- |
|  | Adjusted OR (95% CI) | *P* | Adjusted^*^ OR (95% CI) | *P* |
| Female diver | 3.478 (1.649 – 7.334) | 0.001 | 3.600 (1.503 – 8.623) | 0.004 |
| Age | 1.155 (1.125 – 1.186) | <0.001 | 1.137 (1.085 – 1.192) | <0.001 |
| Diabetes | 2.544 (1.449 – 4.465) | 0.001 | 3.090 (1.032 – 9.257) | 0.044 |
| Hypertension | 2.125 (1.373 – 3.288) | 0.001 | 2.068 (0.563 – 7.601) | 0.274 |
| Cardiovascular disease | 0.331 (0.186 – 0.591) | <0.001 | 0.174 (0.021 – 1.443) | 0.105 |
| Hemoglobin (g/dL) | 0.784 (0.687 – 0.894) | <0.001 | 0.679 (0.488 – 0.943) | 0.021 |
| Serum albumin (g/dL) | 0.748 (0.469– 1.194) | 0.224 | 0.794 (0.231 – 2.727) | 0.714 |
| Total cholesterol (mg/dL) | 1.000 (0.995 – 1.005) | 0.919 | 1.001 (0.991 – 1.011) | 0.833 |

^*^ Adjusted for age, diabetes, hypertension, cardiovascular disease, hemoglobin, albumin, total cholesterol, and breath-holding diving activity.

Abbreviation: eGFR, estimated glomerular filtration rate; OR, odd ratio.
